# Immune function parameters as markers of biological age and predictors of longevity

**DOI:** 10.18632/aging.101116

**Published:** 2016-11-28

**Authors:** Irene Martínez de Toda, Ianire Maté, Carmen Vida, Julia Cruces, Mónica De la Fuente

**Affiliations:** ^1^ Department of Animal Physiology II, Faculty of Biology, Complutense University, Madrid, Spain; ^2^ Institute of Investigation, Hospital 12 Octubre, Madrid, Spain

**Keywords:** immune function, biological age, biomarker, aging, longevity

## Abstract

Chronological age is not a good indicator of how each individual ages and thus how to maintain good health. Due to the long lifespan in humans and the consequent difficulty of carrying out longitudinal studies, finding valid biomarkers of the biological age has been a challenge both for research and clinical studies. The aim was to identify and validate several immune cell function parameters as markers of biological age. Adult, mature, elderly and long-lived human volunteers were used. The chemotaxis, phagocytosis, natural killer activity and lymphoproliferation in neutrophils and lymphocytes of peripheral blood were analyzed. The same functions were measured in peritoneal immune cells from mice, at the corresponding ages (adult, mature, old and long lived) in a longitudinal study. The results showed that the evolution of these functions was similar in humans and mice, with a decrease in old subjects. However, the long-lived individuals maintained values similar to those in adults. In addition, the values of these functions in adult prematurely aging mice were similar to those in chronologically old animals, and they died before their non-prematurely aging mice counterparts. Thus, the parameters studied are good markers of the rate of aging, allowing the determination of biological age.

## INTRODUCTION

The chronological age, defined as the time elapsed since birth, fails to be an accurate indicator of the rate of the aging process [1], which starts at adult age and finishes at the end of the life of each subject. This is due to the heterogeneity that aging shows in the diverse members of a population. This phenomenon led to the concept of “biological age”, which estimates how well an individual functions in comparison with others of the same chronological age [2]. Given that biological age is a better indicator than chronological age of the health, remaining healthy life span and active life expectancy of each subject [2–4], its determination is very relevant. However, despite its simple definition, quantification of the biological age is a difficult task. Many studies have been carried out trying to obtain the most appropriate parameters for determining biological age and have been mainly focused on both physiological (respiratory function, systolic arterial tension, hematocrit…) as well as on biochemical (albumin, cholesterol, blood urea nitrogen…) markers [5–10]. Moreover, other markers such as genetic (telomere length) [11] or epi-genetic (DNA methylation) [12] have also been proposed. Nevertheless, despite different sets of markers being proposed in these studies, none of them have been validated. Therefore, the subject is still incomplete and more research should be carried out.

Most work on biological age has not included parameters of the immune system, which is a homeo-static system that contributes to the appropriate function of the organism. It is well known that with aging there is an increased susceptibility to infectious diseases, autoimmune processes and cancer, which indicates the presence of a less competent immune system, exerting a great influence on age-related morbidity and mortality [13, 14]. Since it has been demonstrated that the functioning of the immune system is an excellent marker of health [15, 16] and given that several age-related changes in immune functions have been linked to longevity [17–19] whereas others have been shown to be predictive of mortality [20–22], the aim of the present study was to determine if some immune functions could be useful as markers of biological age and therefore as predictors of longevity.

In order to validate a potential set of parameters as markers of biological age, it is necessary to confirm that the levels shown in particular subjects reveal their real health and senescent conditions and consequently, their rate of aging. This has to be demonstrated by meeting two requirements. The first is that if an adult individual shows values characteristic of a chronologically old individual, he/she should die prematurely. The second is that a long-lived individual, known to have experienced healthy aging, should have a value of these biomarkers similar to that of an adult [16]. The first requisite can only be confirmed in experimental animals, given that it is a difficult task to follow-up human subjects throughout the whole aging process due to their long life span. Thus, mice were chosen for our study, which show a mean longevity of about 2 years. Previous studies from our group have proposed a murine model of premature aging based on an inappropriate reactivity to stress. Thus, prematurely aging mice (PAM) are identified by their poor response in a simple T-maze test. These PAM at the adult age showed a premature immunosenescence that was accompanied by a shorter lifespan compared to their counterpart non prematurely aging mice (NPAM) of the same sex and chronological age [23, 24]. The second requirement can be confirmed in both humans (centenarians) and experimental animals such as extremely long-lived mice.

Among all the possible functions of immune cells, we have focused on the ones that are the most relevant in the immune response and are known to experience an age-related decrease [25]. In *phagocytes,* their ability to migrate towards the focus of infection (chemotaxis) and their capacity to ingest foreign particles (phagocytosis); in *natural killer (NK) cells*, their capacity to destroy tumor cells and in *lymphocytes*, their ability to migrate towards the site of antigen recognition (chemotaxis) and to proliferate in response to mitogens (lympho-proliferation).

Thus, in order to validate the above mentioned immune functions as markers of biological age and predictors of longevity, these functions were studied in leukocytes isolated from peripheral blood of human subjects in a cross-sectional study, from their 30s until their 100s. In addition, the same functions were analyzed in leukocytes obtained from peritoneum of mice without killing them, enabling a longitudinal study to be performed, starting at the adult age and following each animal until its death. The same functional parameters were also assessed in peritoneal leukocytes from adult PAM and NPAM.

## RESULTS

In this study, in order to identify and validate several functions of immune cells as markers of biological age and predictors of longevity, their age-related changes were measured in leukocytes from both humans and mice.

**Figure 1:**
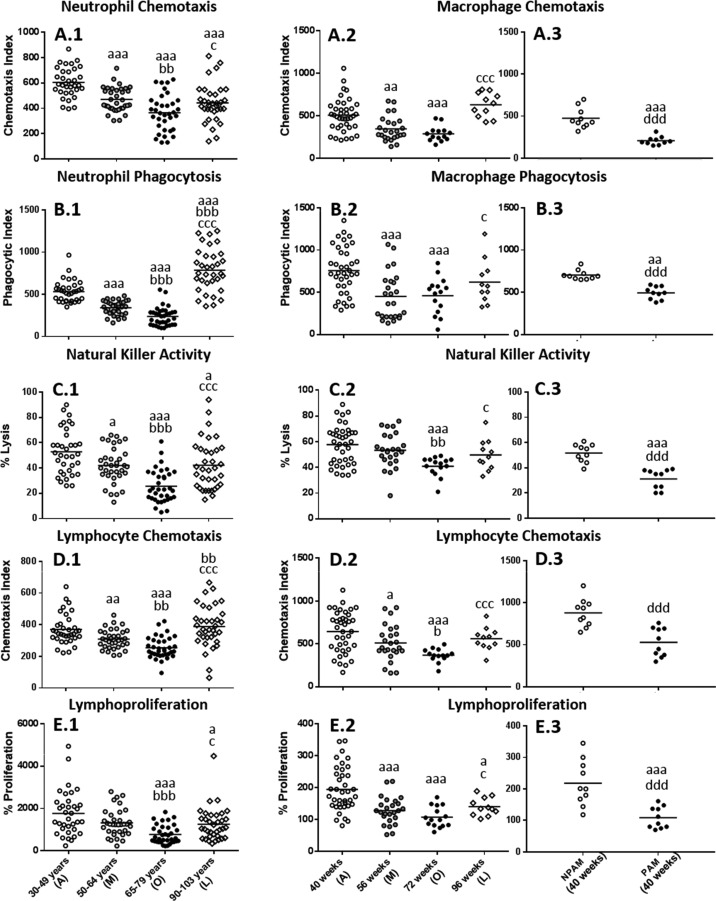
Age-related changes in immune functions in peripheral blood leukocytes from humans and in peritoneal leukocytes from mice (**A**) Phagocytic Index: number of latex beads ingested per 100 human neutrophils (**A.1**) or mouse macrophages (**A.2** and **A.3**); (**B**) Chemotaxis Index: number of phagocytes on the filter, human neutrophils (**B.1**) or mouse macrophages (**B.2** and **B.3**); (**C**) NK cytotoxic activity (percentage of lysis of tumor cells) of human leukocytes (**C.1**) or mouse leukocytes (**C.2** and **C.3**). (**D**) Chemotaxis Index: number of human lymphocytes on the filter (**D.1**) or mouse lymphocytes (**D.2** and **D.3**); (**E**) Percentage of proliferation of lymphocytes in response to the mitogen Phytohaemagglutinin in the case of humans (**E.1**) and in response to Concanavalin A in the case of mice (**E.2** and **E.3**). The results corresponding to peritoneal leukocytes from adult prematurely-aging mice (PAM) and non-prematurely-aging mice (NPAM) are shown in **A.3, B.3, C.3**, **D.3**, and **E.3**. A: Adult; M: Mature; O: Old; L: Long-Lived; a: *P < 0.05*; aa: *P < 0.01*; aaa: *P < 0.001* with respect to the value in adults. b: *P < 0.05*; bb: *P < 0.01*; bbb: *P < 0.001* with respect to the value in mature individuals. c: *P < 0.05*; cc: *P < 0.01*; ccc: *P < 0.001* with respect to the value in old subjects. d: *P < 0.05*; dd: *P < 0.01*; ddd: *P < 0.001* with respect to the value in NPAM.

The results obtained in the functions studied in isolated human blood neutrophils (chemotaxis and phagocytic capacities) and mononuclear cells, principally lymphocytes and NK cells (the anti-tumor cytotoxic activity of NK cells as well as the chemotaxis and proliferative response of lymphocytes to mitogens), obtained from adult, mature, old and long-lived subjects are shown in Fig. 1. Neutrophil chemotaxis (Fig. 1.A1) and phagocytosis (Fig. 1.B1), as well as the activity of NK cells (Fig. 1.C1), lymphocyte chemotaxis (Fig.1.D1) and proliferative response (Fig.1.E1) showed lower values (*P<0.001*) in old individuals in comparison to those in adults. These lower values were also shown in mature subjects (*P<0.001* in neutrophil chemotaxis and phagocytosis; *P<0.01* in chemotaxis of lymphocytes and *P<0.05* in NK activity). In addition, to further explore the relationship between the age and the immune parameters analyzed, *Pearson's* correlations were carried out (Table 1). The results showed statistically significant negative correlations (*P<0.001*) between the age of the subjects and the values of all the immune functions evaluated.

**Table 1:** Pearson's correlation coefficients between the immune functions analyzed in human peripheral blood leukocytes and the age of the subjects

	CORRELATION COEFICIENT (*r*)	*P*
**Neutrophil Chemotaxis**	−0.632	0.000 ***
**Neutrophil Phagocytosis**	−0.711	0.000 ***
**Natural Killer Activity**	−0.589	0.000 ***
**Lymphocyte Chemotaxis**	−0.551	0.000 ***
**Lymphoproliferation**	−0.486	0.000 ***

In peritoneal leukocytes from mice these immune functions, analyzed at the corresponding ages to humans, but in a longitudinal study, changed in a similar manner (Fig.1A2, B2, C2, D2 and E2). Thus, the results showed a significant decrease (*P<0.001*) in the values of all the functions studied at old age with respect to those when they were adults. This decrease was also appreciated at mature age (*P<0.001* in phagocytosis of macrophages and lymphoproliferation, *P<0.01* and *P<0.05* in chemotaxis of macrophages and lymphocytes, respectively). Therefore, the results revealed that these immune functions of murine peritoneal leukocytes present a similar age-related evolution to those in human blood immune cells.

Considering the state of these functions in subjects which reach a high longevity, and consequently have attained successful aging, both humans and mice showed in general, more similar values to those observed at adult age than to those at old age, primarily in mice (Fig. 1). Thus, human centenarians showed higher values in all the functions analyzed with respect to old subjects (*P<0.05* in neutrophil chemotaxis and lymphoproliferation; *P<0.001* in neutrophil phago-cytosis, NK activity and lymphocyte chemotaxis).

Comparing to adults, human centenarians showed a higher phagocytosis (*P<0.001*) but lower neutrophil chemotaxis (*P<0.001*), NK activity and lympho-proliferation (*P<0.05*), whereas no statistically significant differences were found between centenarians and adults in lymphocyte chemotaxis. Long-lived mice also showed a significant increase in all the functions analyzed with respect to when they were old (*P<0.05* in macrophage phagocytosis, NK activity and lympho-proliferation; *P<0.001* in macrophage and lymphocyte chemotaxis). No statistically significant differences were found in mice between when they are adult and long-lived in macrophage chemotaxis and phagocytosis, NK activity and lymphocyte chemotaxis, whereas long-lived mice showed a decrease in lymphoproliferation (*P<0.05*) with respect to the adult age.

**Figure 2:**
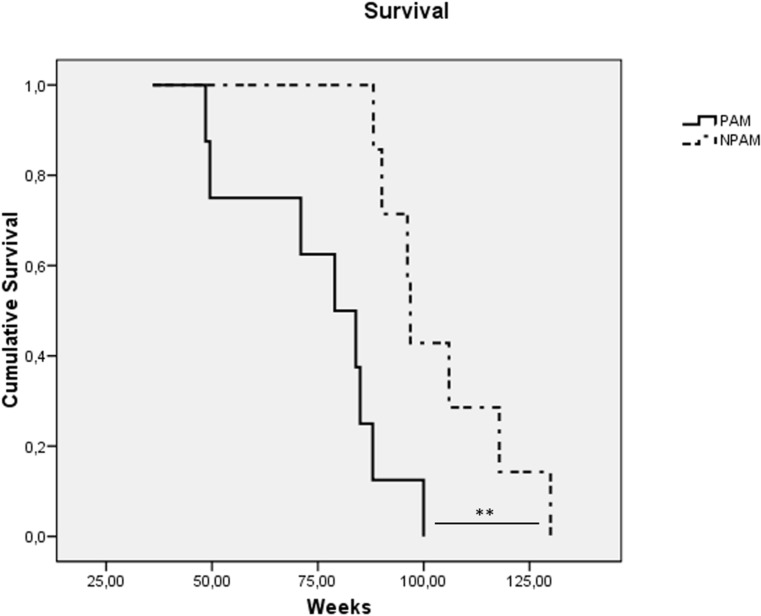
Kaplan-Meier cumulative survival of prematurely aging mice: PAM (solid line) and non-prematurely aging mice: NPAM (dashed line) **: *P < 0.01*.

With regard to the model of premature aging in mice, chronologically adult PAM showed lower values in all the immune functions analyzed (*P<0.001*) in relation to their adult NPAM counterparts (Fig. 1.A3 to Fig. 1.E3). Furthermore, in comparison to adult mice from the longitudinal study, PAM also showed significantly lower values (*P<0.001*) in chemotaxis of macrophages, NK activity and lympho-proliferation, as well as in phagocytosis (*P<0.01*). The values in PAM were similar to those observed in chronologically old mice. Moreover, PAM exhibited a shorter life span (*P<0.01*) than NPAM (Fig. 2).

## DISCUSSION

Many efforts have been made to define the best means for measuring biological age, but considerable disagreement exists concerning the logical strategy for the selection criteria of the corresponding biomarkers. For example, cross-sectional designs can only indicate differences between age groups at a specific point in time. Therefore, in order to conclude that changes have occurred because of aging phenomena, longitudinal designs are needed [7]. In the present study, the selection of biomarkers of aging has been based on both cross-sectional data in humans (because it is very difficult to carry out a longitudinal study throughout aging in our species) and longitudinal data in mice. To our knowledge, this is the first study that combines results from humans and experimental animals as well as showing the results of a longitudinal study performed in mice, initiated at the adult age of 40 weeks (equivalent to 40 years in humans) until the natural death of the animals.

The immune system is relatively immature at birth and completes its development at the end of growth, which is during the second decade of life in human beings [26]. The brain is not fully mature until the early 20s [27, 28] and some hormones reach their peak at 25 years of age [29]. For these reasons, the decision to begin the study at the age of 30 years in humans, and the corresponding age in mice, was taken in order to ensure that all of the adult subjects had already developed a mature and competent immune system and had already begun the aging process.

With respect to the biological age markers chosen, these were relevant functions of the immune system. This physiological system is a complex mechanism of defense of the organism against the continual infectious processes and cancers to which we are submitted since birth. Moreover, this system is also involved in repairing the damage, and maintaining and restoring tissue integrity [30, 31]. To carry out all these functions, the immune system cells, phagocytes and lymphocytes, show a wide range of capacities, which constitute the so-called “immune response”. However, the defensive role of the immune system is compromised during the aging process, since with the advance of age the number of tissue injuries in the body increases as well as the infection processes and cancers and consequently, the mechanisms of defense are more frequently required. In fact, almost every component of the immune system undergoes striking age-associated restructuring, leading to changes that may include enhanced as well as diminished functions. Some of these age-associated changes in immune functions, which constitute the denominated immunosenescence, have been linked to longevity both in mice [25] and in humans [15–18, 32]. In this sense, the immune function parameters are the best choice to be biomarkers of biological age.

Although both innate and adaptive immunity suffer age-related changes, most studies in humans and mice have focused on the severe deterioration of adaptive immune response with age. Thus, aspects of innate immunity most typically studied such as phagocytosis and NK cell cytotoxicity, have been considered to remain relatively unaffected [17]. An aspect of the innate immune response such as mobility to the focus of infection following a chemical gradient (chemotaxis) has been hardly studied in this context. However, more recent work has shown that innate immunity is also very affected by aging [25, 33]. For this reason, and even though a few parameters of adaptive immunity have allowed the definition of the “immune risk phenotype” [19, 34], since they are very limited and have not been validated as markers of biological age, in the present study several relevant functions of innate immunity such as chemotaxis, phagocytosis and NK activity as well as other functions of adaptive immunity such as proliferation of lymphocytes, were chosen. Age-related changes in immune cell populations were not analyzed in this study. Even though the effectiveness of an immune response could be influenced by immune cell population changes (in terms of frequency or counts), the study of these changes could give incomplete information about the immune response capacity. Thus, an increase in the number of a specific immune cell type does not imply a better performance of these cells, but rather the opposite. In fact, it has been shown that there is an age-related increase in the number of CD3-CD56(+) NK cells, which has been explained by a compensatory mechanism towards the age-related loss of the functional capacity of these cells [35, 36].

This is the first study in which these functions have been validated as indicators of an individual's health and biological age. Thus, they were preserved in long-lived individuals (both human centenarians and extremely long-lived mice), which are expected to have withstood the detrimental effects of the aging process better than those individuals who do not live to extreme old age. In addition, mice, which at the adult age already have similar immune function values to those of chronologically old animals, achieve a shorter lifespan. Although this second aspect has not been addressed in humans, since the age-related evolution of these immune functions is similar in both mice and humans, it can be assumed that those humans showing immune parameters at the levels of older subjects have a higher biological age and consequently they will not live so long. In fact, the present study reveals that there is an age-related decline in the immune function parameters analyzed in both human blood neutrophils and lymphocytes, as well as in peritoneal macrophages and lymphocytes from mice, with the lowest values in elderly humans (65-79 years of age) and old mice (72 weeks of age). This age-related decline was further supported by the strong negative correlation coefficients obtained in humans.

Recent studies have suggested that a big cohort of parameters is needed in order to accurately estimate the biological age [37]. The present study demonstrates that the five immune functions analyzed are valid enough to be used as indicators of an individual's health and remaining lifespan and therefore, are proposed as markers of biological age. In addition, these immune function parameters can be useful for the early identification of accelerated aging in humans, which may offer opportunities for prevention of age-related diseases and premature death. Furthermore, as the maintenance of health and consequently the rate of aging, principally depend on environment and lifestyle [16, 38], several strategies such as nutritional (caloric restriction or antioxidant administration) and physical exercise interventions, which can improve the immune response [39], are increasingly being used to slow down the aging process. However, as there is no accurate way of testing their effectiveness, the immune functions proposed in the present study will also be an aid for monitoring the effectiveness of such interventions. Moreover, due to the easy obtainability of the samples in which the immune parameters can be studied, our proposal will be very helpful in controlling health and promoting a healthy longevity.

## MATERIALS AND METHODS

### Subjects

A total of 140 human volunteers (78 women; 62 men) between 30-103 years old were studied and divided into four different experimental groups: adult (30-49 years-old; 18 women and 17 men), mature (50-64 years-old; 18 women and 17 men), old (65-79 years-old; 18 women and 17 men) and long-lived subjects (90-103 years-old; 24 women and 11 men). All the participants are members of the Spanish population. The inclusion criteria were that the subject should be in a healthy condition (absence of pathology or findings of clinical significance in general laboratory parameters). All participants have given written consent for the use of their blood samples for the purposes of this study. All procedures were carried out according to the Declaration of Helsinki.

### Experimental animal

Female ICR/CD1 ex-reproductive mice (*Mus musculus*) were used in this study. Animals were purchased from Janvier Labs (Germany) at the adult age (32±4 weeks), and were placed and acclimatized in the Animal Facility at the Faculty of Biology (UCM). Mice were housed at 4-5 per cage and maintained in standard laboratory animal conditions for pathogens, temperature (22±2°C) and humidity (50-60%), on a 12/12 h reversed light/dark cycle (lights on at 20:00 h) to avoid circadian interferences. Mice had access to tap water and standard pellets (Panlab, Spain) *ad libitum*. One group of animals (n=40) was used for the longitudinal study. The collection of peritoneal suspensions was at the adult (40±4 weeks; n=38), mature (56±4 wk; n=25), old (72±4 wk; n=15) and long-lived (96±4 wk; n=11) ages. Another group of animals were classified as prematurely aging mice (PAM) (n=10) and non-prematurely aging mice (NPAM) (n=10) according to their different behavior in a T-maze, as previously described [23, 24]. After their classification, the collection of the peritoneal suspensions was only at the adult age (40±4 wk). All the experiments were approved by the Experimental Animal Committee of Complutense University of Madrid (UCM) (Spain) and were in accordance with the guidelines of the European Community Council Directives ECC/566/2015.

### Classification of mice according to a model of premature aging: T-maze test

One week after their arrival and acclimation in the Animal Facility of the Faculty of Biology (UCM), one group of adult female ICR/CD1 mice (33±4 weeks) were classified as PAM and NPAM according to their different behavior in a T-maze, as previously described [23, 24]. This T-shaped maze essentially consists of three arms made of wood, whose internal surfaces are covered with black methacrylate. The inside dimensions of each arm are 10 cm wide, 25 cm long, and 10 cm high. The floor is made of 3 mm-thick cylindrical aluminum rods placed perpendicularly to the side walls. The test is performed by holding the mouse by the tip of its tail and placing it inside the “vertical” arm of the maze with its head facing the end wall. Its performance is evaluated with a chronometer to measure the time the animal takes to cross the intersection of the three arms with both hind legs. This test was carried out four times, once a week, to sort the PAM (that required > 10 s to complete exploration of the first arm at each test) from the NPAM (that completed the exploration in < 10 s in each test). Animals showing an intermediate response to the T-maze were removed from the study. Thus, we had two groups of animals: one group including the NPAM population (n=10) and another including the PAM population (n=10). This test was always performed between 09:00 and 11:00 h (to avoid circadian variations) under red light.

### Collection of human blood samples and isolation of neutrophils and lymphocytes

Peripheral blood samples (10 mL) were collected using vein puncture and sodium citrate-buffered Vacutainer tubes (BD Diagnostic, Spain), between 9:00 to 10:00 a.m. to avoid circadian variations in immune para-meters. Neutrophils and lymphocytes cells were isolated from whole blood following a previously described method [40], using 1.119 and 1.077 density Hystopaque (Sigma-Aldrich, Spain) for neutrophil and lymphocyte separation, respectively. Collected cells (95% of viability determined using trypan blue staining) were adjusted to the corresponding final concentrations for the development of each assay.

### Collection of murine peritoneal leukocytes

Murine peritoneal suspensions were collected between 8.00 am and 10.00 a.m., to minimize circadian variations of the immune parameters studied. The use of peritoneal cell sample has the main advantage of not having to sacrifice the animals. Without using anesthesia, mice were held by cervical skin, the abdomen was cleansed with 70% ethanol (Sigma-Aldrich), and 2 ml of sterile Hank's solution, previously tempered at 37°C, was injected into the peritoneum [25]. After massaging the abdomen, approximately the 80% of injected volume was recovered by the needle employed for the injection of Hank's. Leukocytes from peritoneal suspensions were identified by their morphology (macrophages or lymphocytes) and quantified (number of cells/mL) in Neubauer chambers using optical microscopy (x40). Cellular viability, which was routinely checked before and after each experiment by the trypan blue (Sigma-Aldrich) exclusion test, was higher than 95±1% in all cases. The following studies were performed using unfractionated peritoneal leukocytes to better reproduce the *in vivo* situation. The peritoneal suspensions were adjusted to a specific number of macrophages, lymphocytes or total leukocytes, depending on the parameter analyzed as described in the corresponding section.

### Chemotaxis

The induced mobility or chemotaxis of neutrophils, macrophages and lymphocytes was evaluated according to the method previously described [25, 40]. Cell sus-pensions were deposited in the upper compartment of a Boyden chamber separated by a filter of polycarbonate (3 μm of diameter; Merl-Millipore, Ireland). The number of cells (neutrophils, macrophages and lymphocytes) that migrated toward the chemoattractant agent, formyl-Met-Leu-Phe (fMLP, 10^−8^ M, Sigma-Aldrich), deposited in the lower compartment of the chamber, was counted in the lower face of the filter that separates the two compartments of the chamber. This number was expressed as Chemotaxis Index.

### Phagocytosis

Phagocytosis of inert particles (latex beads, 1.1 μm diameter, Sigma-Aldrich) was assayed in phagocytes (neutrophils and macrophages) following a method previously described [25, 40]. Cell suspensions were incubated on migration inhibition factor (MIF) plates (Kartell, Noviglio, Italy) for 30 min at 37°C in a humidified atmosphere. The adherent monolayers obtained were washed with pre-warmed PBS solution, and then 200 μl of Hank's solution and 20 μl of latex beads (1.1 μm diluted to 1% PBS, Sigma-Aldrich) were added. After 30 min of incubation under the same conditions, the plates were washed, fixed with methanol (50%) and stained with Giemsa's solution (Sigma-Aldrich). The number of particles ingested by 100 neutrophils or 100 macrophages was counted and this was expressed as Phagocytic Index.

### Natural killer cytotoxicity

The natural killer (NK) cell cytotoxicity was evaluated following an enzymatic colorimetric assay (Cytotox 96 TM Promega, Boeringher Ingelheim, Germany) based on the determination of lactate dehydrogenase (LDH) released by the cytolysis of targets cells (human K562 lymphoma cells or murine YAC-1 lymphoma cells), using tetrazolium salts [25, 40]. The results were expressed as the percentage of tumor cells killed (% lysis).

### Lymphoproliferation

The proliferation capacity of lymphocytes was evaluated by a standard method, previously described [25, 40]. The assay was assessed in both basal and stimulated conditions using mitogens [phytohema-glutinin (PHA) and concanavaline A (Con A), for humans and mice, respectively]. Cells suspensions were dispensed into 96-wells plates (Costar, Cambridge, MA, USA). 20 μl/well of complete medium, PHA or Con A (1 μg/mL, Sigma-Aldrich) were added. After 48 h of incubation at 37°C in a sterile and humidified atmosphere of 5% CO_2_, 2.5 μCi ^3^H-thymidine (Hartmann Analytic, Germany) were added to each well, followed by another incubation of 24 h. Cells were harvested in a semiautomatic harvester (Skatron Instruments, Norway) and thymidine uptake was measured in a beta counter (LKB, Upsala, Sweden) for 1 min. The results were calculated as ^3^H-thymidine uptake (counts per minute, cpm) for basal and stimulated conditions, and were expressed as lymphoproliferation capacity (%) giving 100% to the cpm in basal conditions.

### Statistical analysis

SPSS 21.0 (SPSS, Chicago, USA) was used for the statistical analysis of the results. All data are expressed as the mean ± standard deviation (SD) of the values corresponding to subjects, being each value the mean of duplicate assays. The normality of the samples and the homogeneity of variances were checked by the Kolmogorov–Smirnov and Levene analyses, respectively. Differences due to age were studied through Student's t test for independent samples. The *Pearson* correlation coefficient was used to test for correlation between parameters. Two-sided *P<0.05* was considered the minimum level of significance. Differences in life span were studied through the Kaplan-Meier test, with a minimum significance level (log rank, Mantel-Cox) also set at *P<0.05*.
